# Efficacy of oral fecal microbiota transplantation in recurrent bowel disease: A protocol for systematic review and meta-analysis

**DOI:** 10.1097/MD.0000000000031477

**Published:** 2022-11-25

**Authors:** Qin Chen, Zhiyun Zhang, Shaosheng Bei, Xiaofeng Wang, Yunying Zhu

**Affiliations:** a Kunming Municipal Hospital of Traditional Chinese Medicine, The Third Affiliated Hospital of Yunnan University of Chinese Medicine, Kunming, China; b Department of Anorectal, Xiyuan Hospital, China Academy of Chinese Medical Sciences, Beijing, China; c Department of Colorectal Surgery, Guang’an men Hospital, China Academy of Chinese Medical Sciences, Beijing, China.

**Keywords:** oral fecal microbiota transplantation, protocol, recurrent bowel disease, systematic review

## Abstract

Recurrent bowel disease (RBD) refers to the chronic, recurrent intestinal diseases, including recurrent Clostridium Difficile Infection (rCDI), inflammatory bowel disease (IBD), irritable bowel syndrome (IBS), etc., these diseases have similar clinical characteristics, that is, abdominal pain, diarrhea, repeated attacks, prolonged recovery, etc. Clinically, there are relevant reports on the use of oral capsule fecal microbiota transplantation (oFMT) to treat RBD. However, both the advantages and disadvantages of clinical efficacy have been reported; there are some contradictions, the study sample size is too small, and the purpose of this systematic review was to evaluate the efficacy and safety of oral capsule fecal microbiota transplantation in the treatment of RBD.

**Methods:**

This systematic review will include articles identified through electronic searches of the PubMed, EMbase, and Cochrane Library. From inception to July 1, 2022. Two reviewers will independently search the database to conduct data extraction and assessment of study quality. Based on heterogeneity tests, data will be integrated using fixed or random effect models. RevMan V.5.4 will be used for data analysis. The results are expressed as the risk ratio of dichotomous data and the mean difference of continuous data.

**Results:**

We analyzed the clinical remission or cure rate, IBS-SSS, quality of life, anxiety, depression, total adverse effects, and total severe adverse effects (TSAE) in patients with RBD.

**Conclusion:**

This systematic review evaluated the efficacy and safety of oFMT in the treatment of RBD to provide more comprehensive evidence.

## 1. Introduction

Recurrent Bowel Disease (RBD) refers to the intestinal chronic, recurrent type of disease, including recurrent Clostridium difficile infection (rCDI), inflammatory bowel disease (IBD), irritable bowel syndrome (IBS), and its common clinical characteristics for chronic or recurrent abdominal pain, diarrhea or stool traits, repeated abnormal symptoms, or even life, seriously affect the patient’s quality of life, and related studies have shown that CDI is associated with disturbances in the gut microbiota.^[1]^ Intestinal microflora dysbiosis plays an important role in the pathogenesis of IBD such as UC.^[2]^ There is evidence of an irregular composition or metabolic activity of the intestinal microbiota in patients with IBS.^[3]^ The intestinal flora disorder and the relative abundance ratio of bacteria imbalance, such as the decrease of probiotics, Escherichia coli, Bacillus, and other pathogenic bacteria increase, can cause various symptoms.^[4,5]^

Fecal microbiota transplantation (FMT) refers to the infusion of fecal suspensions from healthy people into a patient’s gastrointestinal tract to cure a disease by restoring the composition of the intestinal flora.^[6]^ FMT has become a therapeutic option for treating diseases associated with disturbed or depleted gut microbiota.^[7]^ Several reports have shown that FMT is effective in treating diseases associated with gut microbiota bacterial dysregulation, such as CDI, IBD, IBS.^[8]^ rCDI is often associated with microecological imbalance in the intestine caused by antibiotic therapy; however, the recurrence rate is extremely high, and FMT improves the cure rate of recurrent CDI by restoring the diversity of the gut microbiota. FMT is an effective alternative to rCDI treatment and has now been included in standard treatment guidelines in the United States and Europe.^[9]^ Microecological defects and their associated metabolic pathways and molecular mechanisms play an important role in intestinal mucosal innate immunity in IBD, and FMT is a safe and effective regimen for the treatment of IBD. A Meta-analysis showed that FMT was well tolerated in IBD patients, and that the summary estimate of clinical remission achieved in UC patients was 24.1% to 40.5%, compared to 60.5% in CD patients.^[10]^ FMT for IBS reconstructs normal intestinal microflora and its function, thus improving clinical symptoms and quality of life, and altering the gut microbiome by FMT has been suggested as a possible therapeutic option for IBS.^[11]^

FMT can be administered in different ways and at different frequencies with no standard procedures and can be administered through the upper digestive tract (e.g., capsule, nasogastric, nasal duodenum, gastric tube) or colon (e.g., rectum).^[12]^ However, the route of administration via colonoscopic infusion or enema is invasive, poorly tolerated, and logistically difficult owing to storage and transportation requirements.^[13]^ Among other problems, FMT application by colonoscopy or nasal-gastric/duodenal tube exposes the patient to risks and discomfort.^[14]^ A new method of FMT administration involves third-party frozen, encapsulated inoculum that can be delivered orally.^[15]^ Lyophilized FMT contains a large number of bacteria, has stability and survival rates over 3 months after production, and does not require the strict cold chain^[6,7]^ required for FMT freezing.^[16,17]^ It has been shown that patients randomized to receive an FMT enema prefer capsules for future studies.^[18]^ Easy-handling capsules containing donor fecal microbiota provide a safe and efficient treatment for recurrent C. difficile syndrome, suggesting the availability and effectiveness of FMT capsules as a more cost-effective and less invasive treatment.^[9,19]^ There are also related studies on oral FMT for UC and IBS.^[12,13]^

To our knowledge, no systematic review or meta-analysis has focused on oral capsule fecal microbiota transplantation (oFMT) for RBD. Therefore, we aimed to conduct a systematic review and meta-analysis to study the safety and efficacy of oFMT in RBD treatment and to provide methods and evidence for clinical treatment.

## 2. Methods

### 2.1. Study registration

This systematic review and meta-analysis protocol was prepared based on the recommendations of the Preferred Reporting Items for Systematic Review and Meta-Analysis Protocols 2015 statement.^[20]^

### 2.2. Inclusion criteria

#### 2.2.1. Types of studies

All randomized controlled trials (RCTs) of oFMT for RBD will be included in this review. No language limited. Non-RCTs, quasi-RCTs, case series, reviews, and animal studies were also excluded.

#### 2.2.2. Participants

To obtain comprehensive studies and reduce selection bias, we will include all patients with chronic, recurrent intestinal disease, with no restrictions on sex, demographic age, education status, and ethnicity stage.

#### 2.2.3. Interventions

We will include studies assessing and comparing the effectiveness and adverse effects of oFMT for RBD. The route of administration of oFMT includes the upper and lower digestive tract; the duration of treatment and follow-up duration were not limited; the control group included patients receiving a controlled intervention, such as placebo, usual treatment, etc.

#### 2.2.4. Outcomes

The primary outcomes of this review will include the clinical remission or cure rate and IBS-SSS for comparison with oFMT. Secondary outcomes are related to quality of life, anxiety, depression, total adverse effects (such as nausea, bloating, flatulence, abdominal pain, diarrhea, vomiting, dizziness, fever), and TSAE.

### 2.3. Exclusion criteria

1. Described the oFMT only.
2. Conference papers, workshop papers, literature reviews, posters, comments, letters, study protocols, or proceedings papers.

### 2.4. Search strategy

We will use a combination of text words and medical subject headings (MeSH) terms depending on the database to capture the following concepts: RBD and oFMT-based interventions. The following electronic databases will be searched from inception to July 2022: PubMed, EMbase and Cochrane Library. The details of the search strings in the PubMed database are presented in Table 1. Furthermore, the reference lists and bibliographies of relevant studies were manually searched for additional primary studies.

**Table 1:** Search strategy for the PubMed database.

Number	Search queries	Number of hits
#1	“bacteriotherapy feces”[Title/Abstract] OR “donor feces infusion”[Title/Abstract] OR “fecal enema”[Title/Abstract] OR “fecal infusion”[Title/Abstract] OR “fecal matter transplant”[Title/Abstract] OR “fecal microbial transplant”[Title/Abstract] OR “fecal microbiota transplant”[Title/Abstract] OR “fecal transplant”[Title/Abstract] OR “fecal bacterial transplant”[Title/Abstract] OR “fecal bacteriotherapy”[Title/Abstract] OR “fecal enema”[Title/Abstract] OR “fecal instillation”[Title/Abstract] OR “fecal microbe transplant”[Title/Abstract] OR “fecal microbial transplant”[Title/Abstract] OR “fecal microbiome transplantation”[Title/Abstract] OR “fecal microbiota transfer”[Title/Abstract] OR “fecal microbiota transplantation”[Title/Abstract] OR “fecal microflora transplantation”[Title/Abstract] OR “fecal transfusion”[Title/Abstract] OR “fecal transplant”[Title/Abstract] OR “feces bacteriotherapy”[Title/Abstract] OR “feces infusion, donor”[Title/Abstract] OR “feces microbe transplantation”[Title/Abstract] OR “feces microbiota transplantation”[Title/Abstract] OR “feces microflora transplant”[Title/Abstract] OR “fmt “[Title/Abstract] OR “gut microbial transplant”[Title/Abstract] OR “gut microbiome transplant”[Title/Abstract] OR “gut microbiota transplant”[Title/Abstract] OR “gut microflora transplantation”[Title/Abstract] OR “imt “[Title/Abstract] OR “intestinal microbiota transplantation”[Title/Abstract] OR “infusion, donor feces”[Title/Abstract] OR “intestinal microbe transplantation”[Title/Abstract] OR “intestinal microbiome transfer”[Title/Abstract] OR “intestinal microbiome transplant”[Title/Abstract] OR “intestinal microbiota transfer”[Title/Abstract] OR “intestinal microbiota transplant”[Title/Abstract] OR “microbiome transfer, intestinal”[Title/Abstract] OR “microbiome transplant, intestinal”[Title/Abstract] OR “microbiome transplantation, fecal”[Title/Abstract] OR “microbiota transfer, fecal”[Title/Abstract] OR “microbiota transplants, fecal”[Title/Abstract] OR “microbiota transplants, intestinal”[Title/Abstract] OR “rectal bacteriotherapy”[Title/Abstract] OR “stool enema”[Title/Abstract] OR “stool infusion”[Title/Abstract] OR “stool instillation”[Title/Abstract] OR “stool microbial transplantation”[Title/Abstract] OR “stool transplant”[Title/Abstract] OR “transfer, fecal microbiota”[Title/Abstract] OR “transfer, intestinal microbiome”[Title/Abstract] OR “transfer, intestinal microbiota”[Title/Abstract] OR “transplant, fecal”[Title/Abstract] OR “transplant, intestinal microbiome”[Title/Abstract] OR “transplant, intestinal microbiota”[Title/Abstract] OR “transplant, fecal microbiota”[Title/Abstract]	
#2	Fecal Microbiota Transplantation[MeSH Terms]	
#3	“Administration, Oral”[Title/Abstract] OR “Administration, Oral Drug”[Title/Abstract] OR “Administrations, Oral”[Title/Abstract] OR “Drug Administration, Oral”[Title/Abstract] OR “oral drug intake”[Title/Abstract] OR “p.o.administration”[Title/Abstract] OR “Oral Administration”[Title/Abstract] OR “Oral Drug Administration”[Title/Abstract] OR “p.o.dose”[Title/Abstract] OR “p.o.drug intake”[Title/Abstract] OR “per os drug administration”[Title/Abstract] OR “p.o.drug administration”[Title/Abstract] OR “Capsules”[Title/Abstract] OR “capsule, micro”[Title/Abstract] OR “Microcapsule”[Title/Abstract]	
#4	(Administration, Oral[MeSH Terms]) OR (Capsules[MeSH Terms])	
#5	(#1 OR #2) AND (#3 OR #4)	

### 2.5. Study selection

Two trained reviewers independently screened the titles and abstracts of the search results to identify all applicable RCTs. After eliminating duplicate records and ineligible studies, the full text of eligible studies will be reviewed to determine whether they meet the predefined inclusion criteria. Where the researchers are unable to reach a consensus, a third reviewer will make the final judgement.

### 2.6. Data extraction

Two investigators will independently extract information from the included literature and enter the relevant data into a unified data statistics table, including the reference ID, first author, publication year, type of the RBD, patient age, type of intervention, type of control intervention, sample size of each intervention group, intervention time, randomization, allocation concealment and blinding methods, outcome measures, primary outcomes, adverse events, and alteration of intestinal flora, where the reported data are insufficient. The study author will be contacted for further information, and a consensus on data extraction cannot be obtained through negotiation. A third investigator will make the final judgement.

RevMan 5 software (V.5.4; Copenhagen: The Nordic Cochrane Centre, The Cochrane Collaboration, 2014) will be used for data analysis. The use of a fixed-effects or random-effects model will be determined based on the level of heterogeneity. For the 2 categorical variables, risk ratio (RR) and 95% CI will be used. For continuous variables, weighted mean difference or SMD and 95% CI will be used. If there is meaningful heterogeneity that cannot be explained by any assessment (such as a subgroup analysis), no meta-analysis will be performed. If necessary, each subgroup will be carefully considered for subgroup analysis.

### 2.7. Assessment of risk of bias and reporting of study quality

The assessments included random sequence generation, allocation concealment, blinding, incomplete outcome data, selective reporting, and other possible biases. According to the relevant standards in the Cochrane Intervention System Assessment Manual, the Cochrane’s Risk of Bias tool will be used independently by the 2 researchers to assess the quality of the included literature,^[21]^ which will be classified as low, high, or unclear risk. Discrepancies will be resolved through discussions, and consensus will be arrived at with a third investigator, who will make the final judgement where a consensus on risk assessment cannot be reached through discussion.

### 2.8. Data synthesis and analysis

#### 2.8.1. Measures of treatment effect

The effect size was calculated for each study and combined to generate an overall effect size. For results measured on the same scale, the mean difference and 95% CI will be used for effect evaluation, while the standard mean difference (SMD) will be used for results measured on different scales, and dichotomous data will be recorded as risk ratio (RR).

#### 2.8.2. Unit of analysis issues

Data from patients in RCTs will be used. For trials with a crossover design, data from the first sequence will be used. Where multiple non oFMT controls are included, results for all controls will be summarized to analyze the control and intervention groups.

#### 2.8.3. Handling of missing data

For missing data identified during screening and data extraction, the cause of the loss will be determined, and if this is unsuccessful, the missing data will be requested from the study author. If the missing data cannot be obtained, this will be documented and the available data will be extracted and analyzed.

#### 2.8.4. Assessment of heterogeneity

A random-effects or fixed-effects model was used for meta-analysis. According to the Cochrane Handbook for Systematic Reviews of Interventions, heterogeneity can be assessed by a visual check of the forest plot, heterogeneity *χ*^2^ test, and Higgins’ *I*^2^ statistic.^[22,23]^ If the *P* value was >.10, and the *I*^2^ value was <50%, a fixed-effects model was used to pool the data; otherwise, a random-effects model was used. If there is significant heterogeneity between a set of studies, causes of heterogeneity, such as patient characteristics and degree of variation in interventions, will be explored, and sensitivity analysis or subgroup analysis will be used to evaluate heterogeneity if applicable.

#### 2.8.5. Assessment of reporting bias

If more than 10 trials are included in the meta-analysis, a funnel plot will be used to assess the reporting biases, Begg and Egger tests will be used to evaluate the asymmetry of the funnel plot, and values of *P* < .05 were considered to represent significant publication bias.^[24]^

#### 2.8.6. Subgroup analysis

Subgroup analysis was performed according to the heterogeneity of oFMT administration (upper or lower tract), control type (including placebo treatment and conventional treatment), and clinical differences.

#### 2.8.7. Sensitivity analysis

To test the robustness of the review conclusions, a sensitivity analysis was performed for the primary outcome according to the following criteria: sample size, heterogeneity quality, and statistical model (random-effects or fixed-effects model).

### 2.9. Grading of evidence quality

The assessment includes the risk of bias, heterogeneity, indirectness, imprecision, and publication bias, and the quality of the results will be divided into high, moderate, low, and very low.

## 3. Discussion

The long-term recurrent episodes of RBD not only seriously affects the quality of life of patients, but also increases the economic burden of society. The oFMT is a relatively novel treatment method. Although multiple randomized controlled trials of oFMT for RBD have been reported to date, the cumulative evidence for its efficacy has not been systematically evaluated. This study is the first to propose the concept of RBD and to systematically review the safety and efficacy of oFMT in the treatment of RBD. The results provide the evidence of evidence-based medicine.

## Author contributions

**Data curation:** Qin Chen.

**Investigation:** Xiaofeng Wang, Yunying Zhu.

**Methodology:** Zhiyun Zhang, Shaosheng Bei, Xiaofeng Wang.

**Resources:** Shaosheng Bei.

**Supervision:** Qin Chen, Xiaofeng Wang.

**Writing – original draft:** Qin Chen, Zhiyun Zhang.

**Writing – review & editing:** Qin Chen, Xiaofeng Wang.
